# Oral arsenic trioxide for treating acute promyelocytic leukaemia: Implications for its worldwide epidemiology and beyond

**DOI:** 10.3389/fonc.2022.1026478

**Published:** 2022-11-18

**Authors:** Cyrus R. Kumana, Yok-Lam Kwong, Harinder Gill

**Affiliations:** Department of Medicine, School of Clinical Medicine, the University of Hong Kong, Hong Kong, Hong Kong SAR, China

**Keywords:** oral arsenic trioxide (oral-ATO), acute promyelocytic leukaemia (APL), epidemiology, incidence, prevalence, ATO for non-APL diseases (including autoimmune disorders)

## Abstract

This account describes how orally administered Arsenic-trioxide (ATO) therapy influences the epidemiology of acute promyelocytic leukaemia (APL), and how the experience that ensued may expand the indications for oral ATO as a treatment for diseases/disorders other than APL. Over the last two decades, experience with APL patients in Hong Kong treated with an oral regimen comprising ATO, all-trans retinoic acid (ATRA), and ascorbic acid (also known as “AAA”) has confirmed a dramatic improvement in overall survival. Over that period, there has been an estimated 60-fold increase in the prevalence of APL (proportion of surviving APL patients in the population on December 31 including those deemed to be ‘cured’). In contrast to regimens entailing intravenous (IV) ATO, the consequential therapeutic benefits of using oral ATO have been achieved with much less patient inconvenience and quality of life disruption, reduced burdens on health care facilities (hospitalisations and staff involvement), and much enhanced affordability (retail drug & other cost reductions). Numerous experimental and a few clinical studies suggest that ATO may also have a therapeutic role in many other diseases/disorders. Several such diseases (e.g. autoimmune disorders & idiopathic pulmonary fibrosis) are far more prevalent than APL, which means that very large numbers of patients may potentially benefit from ATO treatment, even if its efficacy is limited to selected populations with these diseases. The known safety of oral ATO and its advantages over repeated long-term IV delivery suggests that this route be used in future clinical studies of its possible role in treating such patients. If the clinical utility of oral ATO treatment is validated for patients enduring any such non-APL diseases, very large numbers of patients may stand to benefit.

## How oral ATO treatment has influenced APL epidemiology

Acute promyelocytic leukaemia (APL) is a relatively uncommon type of highly aggressive acute myeloid leukemia, first characterised in 1957 (1–4). However, even till recently − the medical literature contained no authentic estimates for its prevalence, although incidences applicable to various parts of the world were readily available (**Table 1**) (5–9). This seemingly perverse anomaly is very likely due to the very high early mortality of APL treated with conventional chemotherapy,^1^* which has now been superseded by targeted therapy with all-trans retinoic acid (ATRA) and arsenic trioxide (ATO). Paradoxically therefore, prior to this paradigm shift in management, the prevalence of APL could have been lower than its annual incidence.

**Table 1:** Incidence estimates (number of new patients with APL encountered annually, - expressed as a proportion of a given region’s inferred population as cited in relevant publications).

Incidence per 100,000 Person Years	Target Population & Reference	Publication Date & Other Relevant Information
0.15 & 0.17	US^5^	2003: Respective age-adjusted annual rates for females & males are shown, were largely unchanged over successive years (1980−2007). Findings were based on the Cancer Surveillance Program of Los Angeles County.
0.11 ➔ 0.27	US^6^	2012: Based on a Surveillance, Epidemiology, and End Results Database (over 34 years), 1397 patients were identified; rates being 0.11 (in 1975-90) and 0.27 (in 2000-08); in the latter years, ‘better diagnostics’ was offered as a possible reason for the apparent ‘increase’.
0.11 ─ 0.12	Europe^7^	2019: Derieved from a Population based Cross Europe 24 Country study, based on the RARECAREnet database (2000-07). Estimates in different countries varied, were highest in Spain and mostly age-adjusted.
0.37	Hong Kong^8^	2021: Estimate inferred based on 358 newly diagnosed patients over 13 years and assuming an average HK population of 7.5 million during that period.
0.22 ➔ 0.42	Hong Kong^9^	2022: Estimates derived from data available from a Hong Kong APL epidemiology study and yielded a slightly increasing trend (see **Figure 1**).

➔ indicates trend over the years; APL: acute promyelocytic leukaemia.

Even for highly efficacious new treatments with acceptable safety, to become globally established and preferred over alternative more traditional therapeutic strategies ─ they should also be well tolerated, user friendly, convenient/non-disruptive for quality of life, and affordable. In all these respects, treatment of APL patients with oral ATO (prepared in accordance with *Good Manufacturing Practice* standards) confers distinct advantages when compared with intravenous (IV) delivery, which may also be somewhat more cardiotoxic (10–12). Based on these considerations, for more than two decades ─ increasing numbers of newly-diagnosed APL patients in Hong Kong have been treated with oral ATO. Predominantly, such treatment has been incorporated into a regimen comprising ATO, ATRA, and ascorbic acid (AAA) (13). In Hong Kong, patients with APL receiving oral AAA had reasonable quality-of-life with the out-patient regimen and had favourable relapse-free and overall survivals (8–13). Nevertheless, there is lack of prospective data comparing the efficacies and surivals of patients treated with oral ATO-based regimens versus IV ATO/ATRA-based approach.

As to how the prognosis of APL has been transformed, ATO induces dose-dependent apoptosis and differentiation of abnormal promyelocytes *via* its effect on an abnormally generated PML-RARA anti-apoptotic fusion protein, which is involved in a complex series of oxidative processes (14–18). Moreover, its combination with ATRA and ascorbic acid has been shown to produce synergistic degradation of PML in APL cells, whilst also being able to induce apoptosis in myeloma cells (19–24).

**Figure 1:**
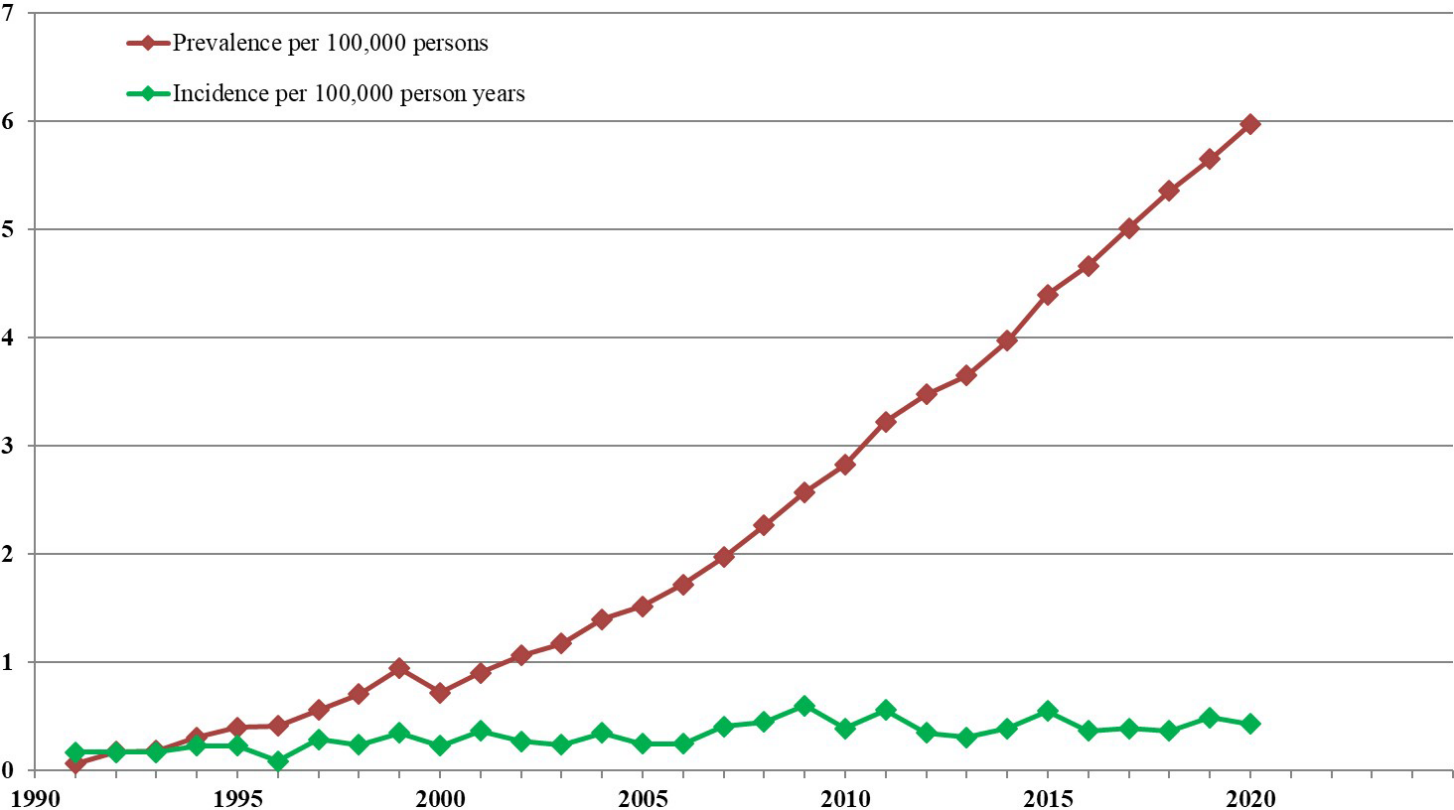
APL Incidence and prevalence in Hong Kong from 1991-2020. For the 18 Hong Kong government hospitals with specialised haematology services, annual incidence and prevalence rates were derived, assuming an average HK population of 7.5 million throughout those years. These estimates were based on available details in the computerised Clinical Data Analysis and Reporting System (CDARS) of the Hospital Authority, Hong Kong. (9) Prevalence refers to numbers of surviving patients diagnosed with APL on December 31 of each year, even if deemed to be ‘cured’. The trend line shows that the point prevalence increased from 0.1 to 6.0 per 100,000 persons between 1991 and 2020.

Thus, assuming that annual incidence rates of APL continue to remain steady, and coinciding with the adoption of highly efficacious treatment with ATRA and ATO (starting around the year 2000) ─ year on year improved outcomes and verifiable increases in prevalence should be expected. This prediction has been amply borne-out, as reported in population based studies of the evolving epidemiology of APL in Hong Kong (9, 25, 26). and illustrated in **Figure 1**, which was constructed from available data as illustrated in **Supplemental Table 1**. The overriding ensuing message is that since around the turn of this century, there has been a marked increase in APL prevalence, related to a dramatic increase in long-term patient survival presumably consequent upon the introduction of treatment with ATRA and ATO. Interestingly, some of the epidemiological publications on APL cited in **Table 1** alluded to increasing year on year incidences (6, 9), whatever the reason.^2^# However, such reported incremental increases were minimal and insufficient to explain the magnitude of prevailing prevalence rate changes. Although it is reasonable to infer that both oral and IV dosing can give rise to equivalent improvements in overall survival and hence increased APL prevalence, the advantages of oral versus IV ATO are nevertheless highly pertinent. This is because intermittent long-term oral ATO administration is easily achieved. In contrast, long-term intermittent IV ATO is much more challenging to implement efficiently as it is less acceptable and burdensome for patients and health care delivery systems in many ways. The impediments specific to IV ATO dosing (10, 27) include being: much more patient unfriendly and restrictive (entailing hospitalisations and absence from work), expensive,^3^‡ highly time consuming, and very demanding on health-care resources.^4^• In contrast, oral ATO can be taken at home under outpatient supervision. In most countries however, reliance on IV dosing still continues to be the norm. This means that APL patients in the poorest parts of the world are needlessly dying, simply because they lack access to oral ATO, an affordable, simple to imbibe, convenient, and quality of life preserving life-saving remedy.

## How experience from treating APL patients with oral ATO could help to expand its indications for the management of patients with non-APL diseases/disorders

Appreciating the favourable impact of a treatment on disease epidemiology, and unravelling the mechanisms for such outcomes, can also have important implications on the potential to expand the indications for such an intervention. The success of ATO therapy, and particularly its oral formulation for APL patients seems to be a case in point, because as of late, there has been a renewed interest in in-vitro, animal, and even clinical studies exploring a possible role for ATO for many other conditions. Thus, there is now a wide array of publications about such non-APL haematological and non-haematological diseases/conditions for which possible ATO-induced treatment benefits are being postulated (28–44). The experimental studies targeted nucleophosmin-1 (*NPM1*)-mutated acute myeloid leukaemia, multiple myeloma, certain lymphomas, lung cancers, several autoimmune diseases, graft versus host disease, certain neurological diseases, and idiopathic pulmonary fibrosis. Preliminary clinical reports also suggest that treatment with ATO may even result in outcome benefits for mantle cell lymphoma and systemic lupus erythematosus (SLE). Compared to APL, moreover, several of the aforementioned non-haematological diseases are much more prevalent and thus clinically important (see **Table 2**) (43–50). Provided the possible putative clinical outcome benefits of ATO for at least some of these conditions are borne out, even for small proportions of such patients, exciting new treatment strategies may well emerge.

**Table 2:** The epidemiology of acute promyelocytic leukaemia and other possible high profile arsenic trioxide responsive diseases.

Disease	Incidence Estimates^†^ (new cases/100,000 person years; see Table 1 for details)	Prevalence Estimates^†^ (Surviving cases/100,000 persons)
APL	0.15 & 0.17 US^5^	- (No values published up to 12/2021)
	0.11 - 0.27 US^6^	- (In earlier decades, prevalence may not have exceeded incidence)
	0.11 - 0.12 Europe^7^	
	0.37 Hong Kong ^8^	
	0.22 ➔ 0.42 Hong Kong ^9^	
SLE	0.7 - 7.4 N America^‡^ } Worldwide^41^ 0.1 - 31.9 Elsewhere^‡^	4.8– 78.5 N America^‡^ } Worldwide^41^ 5.0 - 207.0 Elsewhere^‡^
	? - 23.2 Worldwide^‡,42^	? – 241.0 Worldwide^‡,42^
	3.7 - 7.4 US } Worldwide^43^ 2.9 - 8.6 Elsewhere^‡^	72.8 – 178.0 N America^‡^ } Worldwide^43^ 38.5 - 123.4 Elsewhere^‡^
RA	50.0 South Sweden^44^	660 South Sweden^44^
	40.9 US^45^	720 US^45^
		510 - 560 Worldwide^46^
IPF	0.9 – 13.0 Global^47^	3.3 – 45.1 Global ^47^

APL, acute promyelocytic leukaemia; SLE, systemic lupus erythematosus; RA, rheumatoid arthritis; IPF, idiopathic pulmonary fibrosis. ➔ Trend over time; †Crude, age, and/or gender adjusted estimates; ?, Unrealistic estimates based on very small samples; ‡Very variable depending on ethnicity & location (extremely high in Afro-Caribbeans, and high in Chinese & S Asians) making accurate comparisons challenging.

These considerations are particularly pertinent to the use of oral ATO treatment, as many non-APL diseases entail long-term disability, for which intermittent IV delivery would pose considerable burdens and expense. Thus, the known safety and other advantages of oral ATO^5^*, gleaned from experience treating Hong Kong APL patients, indicate that further clinical studies warrant exploring ATO treatment via the same route for patients having such non-APL disorders and not surprisingly such investigations are being planned. The known likely systemic equivalence of IV and oral ATO means that the latter route offers yet another crucial advantage. Notably, investigations entailing oral dosing offer a means of rejecting or affirming such claims affordably and expeditiously, without imposing quality of life disruption on patients being studied. It also follows that for patients with the latter high prevalence non-APL diseases, robust clinical studies validating worthwhile benefits following oral ATO dosing could confer important benefits for large numbers of chronically symptomatic patients. Improved outcomes for selected patients with such non-APL conditions could also generate heightened interest and support for oral ATO dosing in the medical community and pharmaceutical industry. For diseases that potentially respond to alternative IV arsenicals,^6^† efforts should also be made to determine whether oral ATO may also be effective.

If the findings of such diverse investigations confirm that clinically significant benefits can accrue, very large patient numbers may stand to benefit and thus provide additional clinical and commercial interest in oral ATO.

## Conclusions and future perspectives

Adoption of oral-ATO based treatment for patients with APL in Hong Kong as part of the oral AAA regimen has been associated with vastly improved overall survival and a dramatic increase in estimated prevalence, from about 0.1 to 6.0 per 100,000 persons during the last 2 decades. In contrast to regimens entailing IV ATO, this has been achieved with far less patient inconvenience and quality of life disruption, with greatly reduced direct and indirect costs. In underprivileged parts of the world however, patients with APL are needlessly dying because they lack access to oral ATO. Even in more affluent countries, most patients still continue to receive their ATO intravenously, which imposes unnecessary inconvenience and quality of life disruption, as well as logistical and financial burdens on their health care delivery. For patients with a number of diverse diseases/disorders other than APL, many experimental and a few clinical studies suggest that ATO may also have a therapeutic benefit. Several such diseases are highly prevalent worldwide and give rise to prolonged distress and disability. Hence, robust clinical studies entailing oral ATO in patients with such diseases are obviously warranted. If the expected clinical, logistic and financial benefits for patients with such non-APL diseases can also be validated, very large numbers of patients could stand to benefit. This would very likely rekindle greater interest in this form of ancient therapy within the medical profession and the pharmaceutical industry.

## Author contributions

CRK: Conceived the study, wrote and approved the manuscript. Y-LK: Conceived the study, wrote and approved the manuscript. HG: Conceived the study, wrote and approved the manuscript. All authors contributed to the article and approved the submitted version.

## Funding

The work was supported by the Health and Medical Research Fund (HMRF) (ref.:08191946), Food and Health Bureau, the Government of the Hong Kong Special Administrative Region, China.

## Conflict of interest

Authors HG, CRK and Y-LK are employed by or associated with the University of Hong Kong.

The University of Hong Kong currently holds two United States US patents 7,521,071 B2 and 8,906,422 B2, one Japanese patent 4786341 and one European patent EP 1562616 B1 for the use of arsenic trioxide oral solution in the treatment of leukaemias and lymphomas.

## Publisher’s note

All claims expressed in this article are solely those of the authors and do not necessarily represent those of their affiliated organizations, or those of the publisher, the editors and the reviewers. Any product that may be evaluated in this article, or claim that may be made by its manufacturer, is not guaranteed or endorsed by the publisher.

## Footnotes

1 * Median survival from diagnosis in untreated patients was reported to be less than a week.(3)

2 #Possible speculative explanation being the availability of better ‘diagnostic tools’.

3 ‡One month’s typical IV ATO treatment for an adult with APL retails for around US$10,000; equivalent dosages of oral treatment (Arsenol^®^) used in Hong Kong do so for <40% of this figure, whilst also avoiding the costs and inconvenience of recurring life-long hospitalisations and IV infusion paraphernalia.

4 ∞Repeated time commitments from medical staff and recourse to necessary equipment for IV infusions.

5 *Special circumstances may need consideration; e.g. in chloroquine treated Lupus patients, co-treatment with ATO may risk electrocardiographic QT prolongation related cardiotoxicity.

6
^†^For example, phase I studies in patients with relapsed/refractory Peripheral T-Cell Lymphoma indicate that IV treatment with darinaparsin (an organo-arsenical that leads to cancer cell apoptosis) may confer clinical benefits.(48)
