# Supplementary material for: Oral arsenic trioxide for treating acute promyelocytic leukaemia: Implications for its worldwide epidemiology and beyond
Source: Front Oncol. 2022 Nov 18;12:1026478. doi: 10.3389/fonc.2022.1026478 (PMC9744132; doi:10.3389/fonc.2022.1026478)
Supplement: Supplementary file 1 [file Table_1.docx]

| **Supplementary table. Derivation of Hong Kong APL Prevalence and Incidence Values from 1991-2020** | | | | |
| --- | --- | --- | --- | --- |
| **Year** | **No of patients with APL surviving on 31 Dec** | ***Prevalence per 100,000 persons** | **No. of newly diagnosed patient with APL** | **Annual incidence**  **per 100,000 persons** |
| 1991 | 5 | 0.07 | 13 | 0.17 |
| 1992 | 13 | 0.17 | 13 | 0.17 |
| 1993 | 14 | 0.19 | 13 | 0.17 |
| 1994 | 23 | 0.31 | 17 | 0.23 |
| 1995 | 30 | 0.40 | 17 | 0.23 |
| 1996 | 31 | 0.41 | 7 | 0.09 |
| 1997 | 42 | 0.56 | 22 | 0.29 |
| 1998 | 53 | 0.71 | 18 | 0.24 |
| 1999 | 71 | 0.95 | 26 | 0.35 |
| 2000 | 54 | 0.72 | 17 | 0.23 |
| 2001 | 68 | 0.91 | 28 | 0.37 |
| 2002 | 80 | 1.06 | 20 | 0.27 |
| 2003 | 88 | 1.17 | 18 | 0.24 |
| 2004 | 105 | 1.4 | 26 | 0.35 |
| 2005 | 114 | 1.52 | 19 | 0.25 |
| 2006 | 129 | 1.72 | 19 | 0.25 |
| 2007 | 148 | 1.97 | 31 | 0.41 |
| 2008 | 170 | 2.26 | 34 | 0.45 |
| 2009 | 193 | 2.57 | 45 | 0.60 |
| 2010 | 212 | 2.83 | 29 | 0.39 |
| 2011 | 242 | 3.23 | 42 | 0.56 |
| 2012 | 261 | 3.48 | 26 | 0.35 |
| 2013 | 274 | 3.65 | 23 | 0.31 |
| 2014 | 298 | 3.97 | 29 | 0.39 |
| 2015 | 330 | 4.40 | 41 | 0.55 |
| 2016 | 350 | 4.67 | 28 | 0.37 |
| 2017 | 376 | 5.01 | 29 | 0.39 |
| 2018 | 402 | 5.36 | 28 | 0.37 |
| 2019 | 424 | 5.65 | 37 | 0.49 |
| 2020 | 448 | 5.97 | 32 | 0.43 |
| Dec.: December; no.: number(s).  *Prevalence: Annual no. of APL survivors per 100,000 persons on 31 December of each year  Details pertaining to ever diagnosed APL surviving individuals on 31 December and newly diagnosed APL patients per year were transcribed from the Hong Kong Clinical Data Analysis and Reporting System (CDARS). Prevalence and incidence estimates per 100,000 Hong Kong inhabitants were derived assuming a population of 7.5 million persons. | | | | |
